# Effects of age on foraging behavior in two closely related albatross species

**DOI:** 10.1186/s40462-020-0194-0

**Published:** 2020-02-07

**Authors:** Caitlin K. Frankish, Andrea Manica, Richard A. Phillips

**Affiliations:** 1 British Antarctic Survey, Natural Environment Research Council, High Cross, Madingley Road, Cambridge, CB3 0ET UK; 2 Department of Zoology, University of Cambridge, Downing Street, Cambridge, CB2 3EJ UK

**Keywords:** Aging, Seabird, Senescence, Foraging behavior

## Abstract

**Background:**

Foraging performance is widely hypothesized to play a key role in shaping age-specific demographic rates in wild populations, yet the underlying behavioral changes are poorly understood. Seabirds are among the longest-lived vertebrates, and demonstrate extensive age-related variation in survival, breeding frequency and success. The breeding season is a particularly critical phase during the annual cycle, but it remains unclear whether differences in experience or physiological condition related to age interact with the changing degree of the central-place constraint in shaping foraging patterns in time and space.

**Methods:**

Here we analyze tracking data collected over two decades from congeneric black-browed (BBA) and grey-headed (GHA) albatrosses, *Thalassarche melanophris* and *T. chrysostoma*, breeding at South Georgia. We compare the foraging trip parameters, at-sea activity (flights and landings) and habitat preferences of individuals aged 10–45 years and contrast these patterns between the incubation and early chick-rearing stages.

**Results:**

Young breeders of both species showed improvements in foraging competency with age, reducing foraging trip duration until age 26. Thereafter, there were signs of foraging senescence; older adults took gradually longer trips, narrowed their habitat preference (foraging within a smaller range of sea surface temperatures) (GHA), made fewer landings and rested on the water for longer (BBA). Some age-specific effects were apparent for each species only in certain breeding stages, highlighting the complex interaction between intrinsic drivers in determining individual foraging strategies.

**Conclusions:**

Using cross-sectional data, this study highlighted clear age-related patterns in foraging behavior at the population-level for two species of albatrosses. These trends are likely to have important consequences for the population dynamics of these threatened seabirds, as young or old individuals may be more vulnerable to worsening environmental conditions.

## Background

Aging is ubiquitous in wild vertebrates, with important consequences for population dynamics, and the ecological and evolutionary processes promoting species diversity and co-existence [9, 48, 67]. A range of fitness components vary with age (as reviewed in [67]). These are predicted to explain why survival probability and reproductive success increase in early life, as individuals acquire skills and experience, and decline in old age due to senescence [50, 61, 94]. In reality, the rates, onset, and trajectory of aging often depart from this pattern and vary greatly among and within species [8, 13, 37]. Moreover, the underlying mechanisms are poorly understood, and researching the proximate drivers has become a key topic in the study of aging with wide-ranging implications for life-history theory, population ecology, and wildlife management [53, 60, 88].

Foraging performance is likely to play an important role in shaping the aging process as extracting resources from the environment determines the amount of energy or nutrients animals can allocate to maintenance or reproduction, with consequences for current and future reproduction, and survival [7, 92]. Foraging ability is known to improve in early life, reflecting the development of physical abilities, or the gain in experience of locating and catching prey [39, 41, 113]. Acquiring these skills can directly improve survival probability, and foraging performance can continue improving past sexual maturity as animals learn to adapt to the added constraints of breeding [31]. Evidence for age-related variation in foraging behavior in later life is rarer, and more difficult to interpret. Differences between old and young adults in activity budgets, diets, distribution, habitat use and other foraging characteristics have been linked to physiological declines [19, 58, 62], with consequences for fitness in some instances [21, 42, 52]. However, changes in foraging behavior with age may not be detectable if individuals are able to compensate for physiological aging, warranting further investigation across multiple taxa [33, 76].

Seabirds, and albatrosses in particular, are excellent models for studying aging as they are among the longest-lived vertebrates, with some individuals reaching over 60 years of age [100, 101]. Long-term monitoring studies demonstrate considerable age-related variation in their reproductive performance [36, 69, 71], and remote-tracking techniques provide effective tools for investigating their foraging behavior [20, 49, 111]. Albatrosses cover remarkable distances while foraging at sea, but their energetic requirements and reproductive demands change throughout the year, limiting foraging in time and space to different extents [76, 102]. The breeding period is an especially critical phase during their annual cycle, as individuals are under strong selection to forage efficiently in order to relieve fasting partners during incubation, and to feed both themselves and their young during chick-rearing [76]. Inexperience may be a constraint in young breeders if they are less-skilled at acquiring prey items [43, 51, 64]. Reduced physiological condition in older breeders may have a similar effect, manifested as extended foraging trips, reduced foraging effort, or differential habitat use in the few seabird studies to date [18, 46, 52]. As these findings largely relate to analyses from a single breeding stage, it remains unclear however *how* these intrinsic attributes interplay with the changing degree of the central-place constraint in shaping foraging patterns in time and space. Investigating this question will provide crucial insight into the ecological forces shaping aging trends and driving the population dynamics of this highly threatened group of seabirds [75].

Here we performed a cross-sectional study to investigate the links between age, foraging behavior and breeding stage in grey-headed and black-browed albatrosses, *Thalassarche chrysostoma* and *T. melanophris* (hereafter GHA and BBA, respectively) tracked from Bird Island, South Georgia, between 1997 and 2015. GHA and BBA are closely-related, similar in size and breeding cycle but differ in aspects of their life-history strategies (breeding frequency, lifespan and age-specific breeding success, [11, 36, 78, 80]). In particular, only in GHA are there signs of senescence in reproductive success [36]. This accords with some evidence of longer trip durations and reduced foraging efficiency in older breeders during incubation [19]. Here, we build on that initial tracking study by incorporating movement and activity data from multiple breeding stages and study years for both GHA and BBA, to investigate whether species-specific aging trajectories may be driven by differences in foraging behavior. Specifically, we hypothesize that young adults of both species may have reduced foraging competency, and therefore take longer trips to less-productive areas, and have a higher take-off and landing rate, as they may be less skilled at finding or handling prey. As only GHA show signs of reproductive senescence, we hypothesize that only this species will show signs of foraging senescence, by taking longer foraging trips, and spending a larger proportion of these trips resting on the water as a result of physical deterioration. For the same reasons, we expect old GHA to differ from younger birds in habitat use, targeting less productive or more accessible foraging areas [98]. Finally, we contrast these patterns between breeding stages, expecting age effects to be more pronounced during incubation when the central-place constraint is less severe and individuals conduct long-range trips [78]. We also expect age effects to differ between sexes, given the degree of sexual dimorphism in wing loading and wing area, and evidence for spatial segregation in these species during the early breeding season [78].

## Methods

### Tracking data

Tracking data used in this analysis were collected from GHA and BBA on Bird Island, South Georgia (54°00′S, 38°03′W), during the austral breeding seasons between 1992/93 and 2014/15 (for deployment details, see Phillips et al. [78]; Phalan et al. [74]; Scales et al. [90]). Hereafter, each breeding season is identified by the year in which the chicks fledge, e.g. 1992/93 as 1993. Locations were recorded using GPS loggers and Platform Terminal Transmitters (PTTs), with the mean interval dependent on GPS scheduling and number of fixes provided by the ARGOS satellite system (Additional file 1: Table S1). Typically, birds with PTTs were also fitted with a 17 g radio transmitter attached to a plastic band on one tarsus which allowed exact arrival and departure times to be determined using a remote radio-receiver logger system (Televilt); otherwise, these were estimated from satellite fixes and visual observations during nest visits. In all cases, the total mass of devices including attachments was less than the 3% threshold of body mass beyond which deleterious effects are more common in pelagic seabirds [79].

Chicks have been ringed annually since the 1970s, and the majority of the population in intensive study colonies on Bird Island is of known age. The sex of all birds (or their partners) was either determined from records of observed copulatory position, pre-laying attendance pattern, or using DNA extracted from a blood sample [34] . Birds of known sex but unknown age were assigned a conservative minimum age of 8 years (BBA) or 10 years (GHA) when first ringed as breeding adults [95]. Trips by these particular birds were only included in the analysis if their age when tracked exceeded the average age at which senescence in breeding success is apparent in the study populations [36].

Individual trips were processed using an iterative forward/backward-averaging filter to remove any locations which required sustained flight speeds above 90 km.h^− 1^ [58]. Seven additional locations missed by the filter were later removed following visual examination of the tracks. Five tracks were incomplete because the device battery failed during the trip. Visual inspection indicated that this occurred during the outward portion of the trip in three instances, and during the return trip in two others. The former were excluded as no trip metrics could be calculated, and the latter were deemed ‘near-complete’ and included in further analyses. Finally, one trip that lasted for less than 6 h was also excluded as it is likely that the adults were close to the colony and did not forage during that time [79, 106].

As different devices and scheduling were used in different years (Additional file 1: Table S1), the processed tracks were interpolated to 30 min intervals (close to the mean for all recorded trips) using function ‘redisltraj*’* in package ‘adehabitatLT’ [14]. As very few individuals of known age (7%) were tracked for multiple trips, one trip was chosen at random for those birds. Data from the post-brood chick-rearing stage were excluded as the sample size for birds of known or minimum age was insufficient for further statistical analysis (4 trips). The final sample size was 51 tracks from the incubation stage (35 BBA and 16 GHA) and 107 tracks from the brood-guard stage (69 BBA and 38 GHA), collected between 1997 and 2015 from birds ranging between 10 and 45 years of age.

Immersion data were available in 2002, 2008, 2010 and 2015 for BBA and in 2003, 2010 and 2012 for GHA. These were collected using loggers with two different sampling protocols. Lower-resolution loggers (Mk IIa-V; British Antarctic Survey [BAS]) tested for saltwater immersion every 3 s, storing the sum of positive tests every 10 min as a value ranging from 0 (continuously dry) to 200 (continuously wet). Higher-resolution loggers (GLS C-250 Intigeo; Migrate Technology Ltd., Cambridge, UK) also tested for immersion every 3 s, but recorded the time of transition between wet/dry states that lasted ≥6 s, providing the timing and duration of flights and landings, and consequently a more accurate indication of albatross activity throughout a given subset of foraging trips. Data from both loggers were used to calculate the proportion of the trip spent dry (in flight) versus wet (on the water). Immersion data were matched to corresponding GPS and PTT locations, providing data on at-sea activity for 44 tracks from the incubation (29 BBA and 15 GHA) and 86 tracks (54 BBA and 32 GHA) from the brood-guard stage. All data manipulations and analyses were conducted in R ver. 3.5.1 [81].

### Trip characteristics and activity pattern analysis

Depending on data availability, the following metrics were calculated for each foraging trip: (1) trip duration (days); (2) maximum range (maximum distance reached from colony in km), calculated using function ‘homedist*’* in package ‘trip’ [93], (3) latitude at maximum distance from colony, (4) landing rate (wet events per hour), calculated as the total number of wet-dry transitions, (5) mean wet bout duration (minutes), and (6) wet time (proportion of total trip spent on the sea surface). Variables (4) and (5) were only available from high-resolution loggers. Variables (4), (5) and (6) were calculated separately for daylight and darkness as these albatross species are predominantly diurnal feeders [74], using the function ‘crepuscule*’* in package ‘maptools’ to determine the timing of civil twilight (when the sun is 6 degrees below the horizon, [6]). ‘Day’ (daylight including twilight) or ‘Night’ were assigned accordingly. As there were only high-resolution immersion data for six GHA, metrics (4) and (5) were only investigated in BBA.

The relationships between these metrics, and age (‘Age’), sex (‘Sex’), species (‘Species’) and breeding stage (‘Stage’) of the birds, as well as the two-way interactions were investigated using linear models. ‘Age’ was modelled as a continuous variable, and each model tested for both linear and quadratic relationships between age and the various metrics to approximate the relationship previously found between age and breeding success at the population level in BBA and GHA [36]. The models included two-level factors for ‘Sex’ (Male and Female), *‘*Species’ (BBA and GHA) and ‘Stage’ (Incubation and brood-guard). Study year (‘Year’) was also included as an additive fixed effect to account for annual variation in environmental conditions, and was modelled as a seven-level factor for metrics (1)–(3) (1997, 2002, 2003, 2008, 2010, 2012, 2015), a three-level factor for metrics (4)–(5) (2008, 2010, 2015), and a six-level factor for metric (6) (2002, 2003, 2008, 2010, 2012, 2015). Metric (1) was square-root transformed, metrics (2), (4) and (5) were log-transformed, and metric (6) was logit-transformed to improve data spread. All possible models were ranked according to Akaike Information Criterion (AICc) values, and the most supported model(s) were considered as all models within 2Δ AICc of the top model [12]. Candidate models were excluded from this set if they were more complex variations of other candidate models with lower ΔAICc values [3]. We did not consider models that contained age as a quadratic but not linear term (Age^2^ without Age), or the interaction of the quadratic but not the linear age term with another linear predictor (e.g. Age^2^: Stage without Age: Stage) for the models to remain well-formulated [5, 73]. To prevent overfitting, all possible models were ranked in a second instance according to Leave One Out Cross Validation (LOOCV), and the top models were compared with those ranked according to AICc values [54].

### Behavioural classification

Landings derived from immersion data are often used to identify foraging bouts in albatrosses [74, 90], as take-offs are energetically costly, and immersion events are likely to indicate prey capture attempts [91]. As immersion data were not available for all trips, the Expectation Maximization binary Clustering (EMbC) algorithm was used to identify foraging bouts that were modelled in the subsequent habitat analysis. EMbC is a robust, non-supervised multi-variate clustering algorithm leading to meaningful local labelling of tracking locations based on the speed and turning angle obtained from successive locations [38]. The population-level analysis tool ‘binClstStck’ was used to analyse all tracks, and locations were classified according to four different clusters of high (H) and low (L) values of speed and turning angle. Clusters 2 and 4 were merged, grouping both low and high speeds at high turning angles (LH and HH), and resulting clusters were interpreted as follows: (1) LL as ‘Resting’, (2) LH and HH as ‘Foraging’, and (3) HL as ‘Transit’ (following [57]). The plausibility of the EMbC behavioral clustering was verified by summarizing the landing rate and wet time during each state for all trips with immersion data (Additional file 1: Figure S1 and Table S2).

### Habitat preferences and oceanographic data

The habitat preferences of tracked BBA and GHA were investigated by comparing the environmental characteristics at the locations of foraging bouts with those in the areas that were available (use-availability) using binomial generalized additive models (GAMs), which allow for non-linear relationships between animals and the environment [1, 109]. Available areas were determined by generating 50 time-matched pseudo-absence points for every foraging bout location classified using EMbC by randomly rotating the foraging bout location around the study colony (Bird Island) to take movement constraints into account [99]. Pseudo-absences were re-generated if they intersected with land.

Environmental predictors (summarized in Table 1) were selected as proxies of oceanographic and topographic features known, or hypothesized to be of importance for habitat selection in pelagic seabirds [43, 77, 90, 99, 111]: (1) ocean floor depth (DEPTH - indicative of productive bathymetric areas such as shelf-breaks, seamounts and upwelling, [40]), (2) sea surface temperature (SST - indicative of water masses, [68, 83]), (3) chlorophyll α concentration (CHL - indicative of primary productivity, [24]), (4) eddy kinetic energy (EKE), and (5) sea level anomaly (SLA), indicators of mesoscale turbulence [25], (6) wind speed (WIND - linked to movement costs and prey availability, [66, 114]). All environmental datasets were accessed in 2018. Three further variables were calculated using function ‘focal’ in package ‘raster’: (7) depth slope (DEPTH SD; indicative of topographic features), (8) SST gradient (SST SD; a proxy for thermal fronts), (9) Chl gradient (CHL SD; another proxy for fronts), and (10) tracking year was included as a fixed effect (‘Year’). All variables were downloaded as daily composites and resampled to 0.25°, corresponding to the coarsest scale of all datasets; using bilinear interpolation, recommended for continuous data [70]. All environmental data as well as the location data were projected using the Lambert Conformal Conic projection centered at 37°W and 54°S (EPSG:3762), to limit distortion. Mean covariate values at the location of each foraging bout and pseudo-absence were then extracted using a 1.5 km buffer with the function ‘gBuffer*’* in package ‘raster’ to account for PTT location error [22]. Locations with missing environmental values due to gaps in satellite observations (usually of wind speed) were excluded, resulting in a minimum of 47 pseudo-absences per foraging-bout location. The four tracks from the breeding season of 1997 were not included in further habitat analysis as chlorophyll data were not available.

**Table 1:** List of variables used in habitat analysis

Variable	Abbreviation	Source	Temporal resolution	Spatial resolution
Bathymetry	DEPTH	GEBCO		0.02°
Bathymetric gradient	DEPTH SD	Calculated as standard deviation of Depth using function ‘focal’ in package ‘raster’		0.02°
Sea surface temperature	SST	NOAA OI SST V2 High-resolution blended dataset	1 day composite	0.25°
Sea surface temperature gradient	SST SD	Calculated as standard deviation of SST using function ‘focal’ in package ‘raster’	1 day composite	0.25°
Eddy kinetic energy	EKE	Copernicus global ocean gridded L4 sea surface heights and derived variables reprocessed	1 day composite	0.25°
Sea level anomaly	SLA			
Wind speed	WIND	NOAA blended sea winds	1 day composite	0.25°
Chlorophyll a concentration	CHL	Copernicus global ocean chlorophyll L4	1 day composite	0.04°
Chlorophyll a concentration gradient	CHL SD	Calculated as standard deviation of Chl using function ‘focal’ in package ‘raster’	1 day composite	0.04°

Predictor variables were checked for collinearity by calculating all pairwise Spearman rank correlation coefficients. CHL and CHL SD were highly correlated (> 0.6), and so two models were run with each predictor and compared using AIC. The model with CHL resulted in the lowest AIC value, and thus was interpreted as the better fit.

Separate models were constructed for different classes of birds because of computational demands and difficulties of interpreting high-order interactions. Initial models testing for interactions between species and breeding stage were significant, so the full model was split into four components, by species (BBA vs. GHA) and breeding stage (Incubation vs. Brood-guard). Using methods similar to Žydelis et al. (2011), the effect of different numbers of pseudo-absences was tested on the performance of these four models. Each individual model contained smoother splines for the environmental variables as well as for interaction of these variables with age. Smoothers were produced using cubic regression splines with shrinkage which penalize variables during fitting to reduce over-parameterization, and *k* was set to a maximum of 4 knots to reduce over-fitting [109]. A set of models consisting of all observed tracks and varying numbers of simulations (up to 47) per individual found that both the χ^2^ for each parameter and the area under the receiver operator curve (AUC) stabilized around 20–30 pseudo-absences per individual. Consequently, 30 pseudo-absences per observed track were chosen for subsequent analysis (Additional file 1: Figure S2).

The inclusion of a random intercept for individual ID can help control for variability in response to the environment; however, model selection and inference in large datasets are computationally demanding within the mixed effects framework [1, 109]. The best minimal models were thus determined by forward selection using *k*-fold validation, testing the goodness of fit of each individual, in turn, against the prediction based on the other individuals [15, 99]. Model selection was based on the predictive ability of the models using Area Under the Curve (AUC) averaged across the *k* sets of results (i.e. individuals) using the ‘pROC’ package [82, 87]. AUC values of 0.5–0.7, 0.7–0.9 and > 0.9 represent poor, reasonable and very good model performances, respectively. The forward selection procedure consisted initially of fitting all possible single environmental predictors with and without the age-interaction and ranking these models according to AUC. The best ranking model was chosen, and then each of the remaining predictors was added in turn (with and without the age interaction) and the best model among this new set was then retained if the AUC increased significantly. This process was repeated until there was no longer a significant increase in AUC between two models based on paired *t*-tests. Habitat preference models were fitted separately for the incubation and brood-guard stages for both BBA and GHA.

## Results

**Fig. 1:**
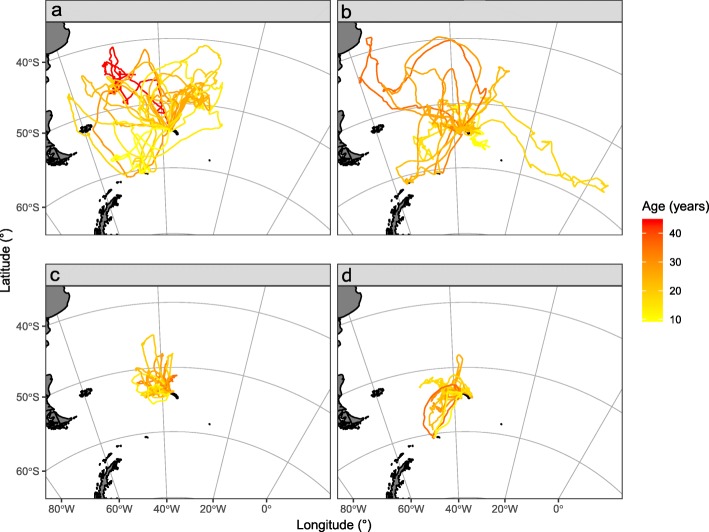
Distribution of foraging trips from all aged and sexed black-browed albatrosses breeding at Bird Island, South Georgia, during the incubation and brood-guard stages in austral summers 1996/97 to 2014/15. **a** incubating females (17 tracks), **b** incubating males (18 tracks), **c** brood-guard females (20 tracks) and **d** brood-guard males (49 tracks)

**Fig. 2:**
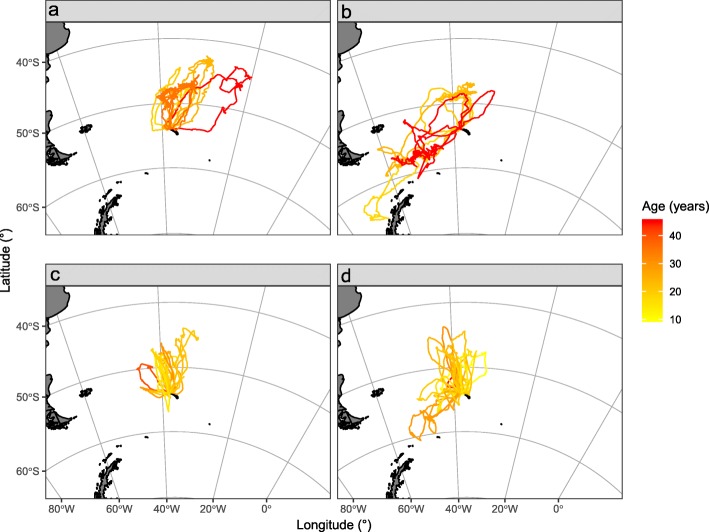
Distribution of foraging trips from all aged and sexed grey-headed albatrosses breeding at Bird Island, South Georgia, during the incubation and brood-guard stages in austral summers 2002/03 to 2011/12. **a** incubating females (9 tracks), **b** incubating males (7 tracks), **c** brood-guard females (13 tracks) and **d** brood-guard males (25 tracks)

Tracked BBA and GHA foraged over a wide area around Bird Island during the incubation and brood-guard stages (ranging from 38 to 65°S and 73°W-5°E; Figs. 1 and 2), and showed age-related variation in foraging trip characteristics, activity patterns and habitat preferences (See Additional file 1: Tables S3 and S4, for full model selection and parameter estimates).

### Age-related variation in trip characteristics

**Fig. 3:**
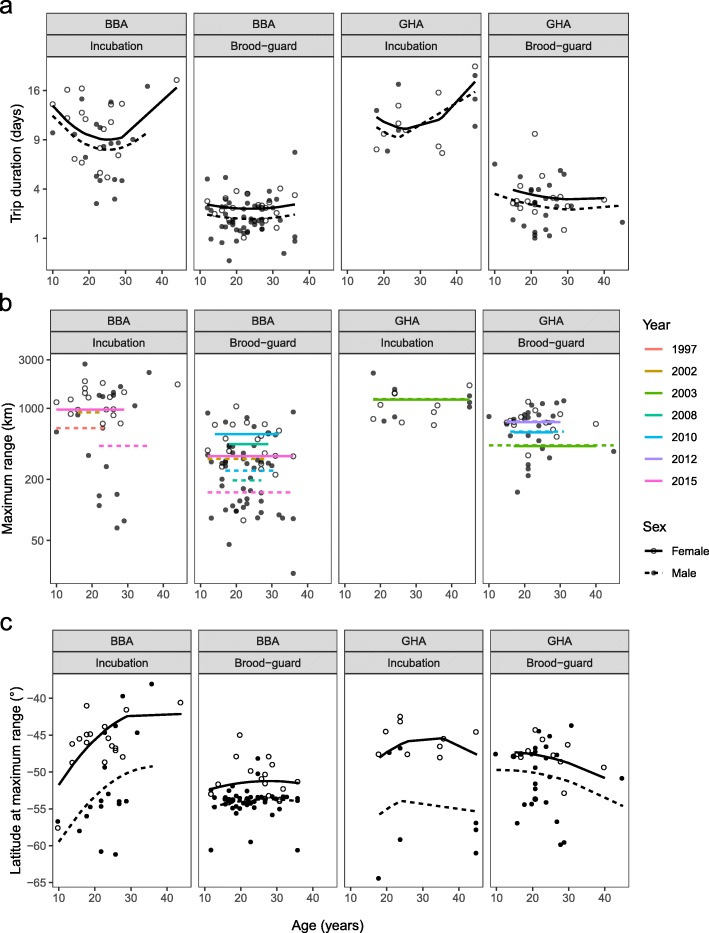
Relationship between age and foraging behaviour for male (closed circles) and female (open circles) black-browed (BBA) and grey-headed (GHA) albatrosses during the incubation and brood-guard stages (**a**-**c**). Regression lines indicate the fitted values of the average of the most supported models for each response variable. Where a significant effect of sex was found, males (dotted) and females (solid) are shown with separate lines. Horizontal lines indicate no age effect but a significant sex effect. Values of transformed response variables (**a** and **b**) are back-transformed on the y-axis but the scale of the transformation is retained

The age of BBA and GHA had a strong effect on the duration of their foraging trips, as evidenced by the age terms (Age, Age^2^, Age: Stage, Age^2^: Stage and Age: Species) retained in the average of the top models (Table 2, Fig. 3a). During the incubation stage, the duration of foraging trips of both species declined in early adulthood until age 26 years (BBA: modelled change of − 4.3 [36%] and − 4.6 [34%] days in males and females respectively, GHA: modelled change of − 1.4 [13%] and − 1.6 [13%] days in males and females respectively), although this relationship was not as pronounced in GHA because fewer young birds were tracked during incubation (only 6 GHA were < 26 years and all 6 were ≥ 18 years). Foraging trip duration then increased in both species as the birds reached old age (BBA: modelled change of + 2.1 [26%] and + 7.5 [83%] days in males and females, respectively, GHA: modelled change of + 6.6 [71%] and + 7.1 [69%] days in males and females, respectively). Although this trend may be driven in BBA by the two oldest birds, the top two models ranked according to LOOCV contained the same predictor variables as those ranked according to AICc, suggesting outliers had little influence on model selection (Additional file 1: Tables S3 and S5). The quadratic relationship with age was less apparent during the brood-guard stage, when mean trip durations were considerably shorter (by ~ 7.6 days). Overall, GHA took slightly longer trips on average than BBA (by ~ 1.0 days), and females took slightly longer trips than males regardless of species and stage (by ~ 2.2 days).

**Table 2:** Effects of age (Age = linear relationship, and Age^2^= quadratic relationship), sex, stage, species and year on trip characteristics and activity patterns of black-browed and grey-headed breeding at Bird Island, South Georgia. ‘x’ indicates terms retained in the average of the best-supported models for each response variable (full model selection and parameter estimates are listed in Additional file 1: Tables S3 and S4)

Response variable	n	Predictor variables															
		Intercept	Age	Age^2^	Sex	Stage	Species	Year	Age: Sex	Age^2^: Sex	Age: Stage	Age^2^: Stage	Age: Species	Age^2^: Species	Sex: Stage	Sex: Species	Stage: Species
Trip duration (days)	158	x	x	x	x	x	x				x	x	x				
Max range from colony (km)	158	x			x	x	x	x								x	
Latitude at max range (°)	158	x	x	x	x	x	x				x	x	x		x		x
Landings.hr.^−1^ in daylight^a^	66	x	x		x			x	x								
Landings.hr.^−1^ in darkness^a^	64	x						x									
Wet bout length in daylight (mins)^a^	66	x	x		x												
Wet bout length in darkness (mins)^a^	64	x						x									
Prop daylight wet (%)	130	x					x	x									
Prop darkness wet (%)	128	x			x	x	x	x									

^a^ Species was not included in the model for these two metrics as sample size was very small for GHA

Age was also included as a quadratic term in the top models explaining the latitude reached by birds at maximum distance from the colony (Table 2, Fig. 3c), suggesting age-related segregation in foraging distributions, and warranting further investigation of habitat preferences. Incubating BBA foraged at progressively northerly latitudes with increasing age (increase in 10.2° of latitude in males aged between 10 and 36 years and in 9.6° of latitude in females aged between 10 and 44 years). GHA during incubation showed very little age-related variation in latitude but foraged at progressively southerly latitudes with increasing age during the brood-guard stage (decrease in 4.9° of latitude in males aged between 10 and 45 years, and 3.5° of latitude in females aged between 15 and 40 years), whereas BBA of different ages foraged at similar latitudes during brood-guard, usually close to Bird Island between − 55° and − 56° S (Fig. 3c). Overall, females foraged at more northerly latitudes (by ~ + 4.6°) than males, especially during incubation (Fig. 3c; the difference in the latitudes reached by females and males increased during the incubation stage by ~ 4.4°). GHA foraged on average at more northerly latitudes than BBA, especially during the brood-guard stage (by ~ + 3.5°).

Age did not, however, influence the maximum range of birds during foraging trips (Table 2, Fig. 3b). As expected, all birds foraged further afield during the incubation stage (by ~ 517 km on average). Male BBA foraged on average 392 km closer to the colony than female BBA regardless of stage, but there was less difference (~ 143 km) between the maximum ranges of male and female GHA. This metric also varied significantly between study years; by 523 km and 305 km between the lowest and highest average yearly ranges for BBA and GHA, respectively.

### Age-related variation in activity patterns

**Fig. 4:**
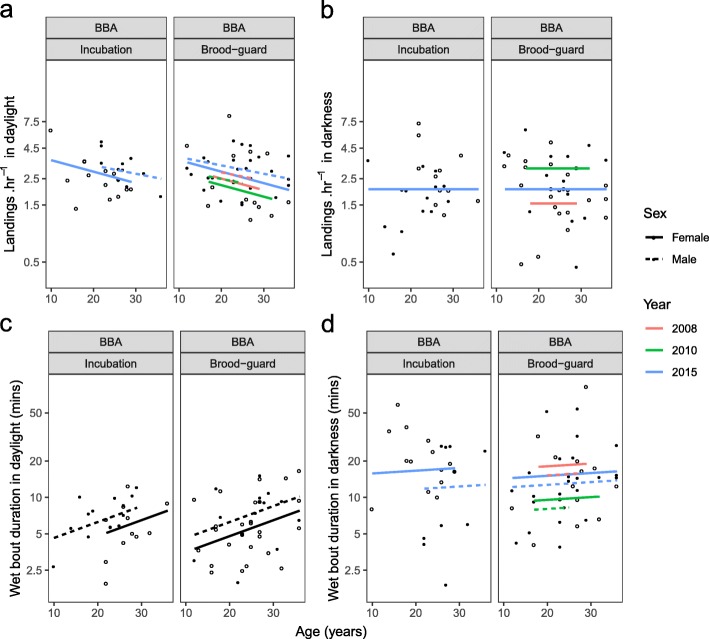
Relationship between age and high-resolution activity metrics for male (closed circles) and female (open circles) black-browed (BBA) albatrosses during the incubation and brood-guard stages. Regression lines indicate the fitted values of the average of the most supported models for each response variable. Where a significant effect of sex was found, males (dotted) and females (solid) are shown with separate lines. Horizontal lines indicate no age effect but a significant sex effect. Values of transformed response variables (**a**-**d**) are back-transformed on the y-axis but the scale of the transformation is retained

Age was retained in the top models describing landing rate and mean wet bout duration of BBA in daylight (Table 2, Fig. 4a and c). With age, BBA landed less often on the water (modelled change of − 1.2 landings.hr.^− 1^ [32%] and − 1.6 landings.hr.^− 1^ [44%] between 10 and 36 years old for males and females respectively; Fig. 4a). The third most-supported model for this metric suggested a faster decline in landing rate with increasing age in female BBA, but this effect was deemed minimal as it was only included in one of the top three models (Additional file 1: Table S3). BBA also spent increasing time on the water between landings (modelled change of + 3.2 min [55%] between ages 12 and 36 years, and + 5.5 min [52%] between ages 10 and 36 years for males and females, respectively). This trend was apparent for both breeding stages, but females spent slightly more time on average on the water in daylight than males (by 1.4 min). Age, however, had little bearing on these metrics during darkness. Instead, mean landing rate and wet bout duration in darkness varied strongly between study years (Table 2). BBA were the least active in darkness in 2008, landing less often (by ~ 1.5 landings.hr.^− 1^) and spending more time on the water between landings (by ~ + 7.8 min) than in 2010, the year when activity was highest. Age, stage and sex effects were included in the third top model explaining variation in wet bout duration during darkness, but as these terms were not included in the other two models, their effects were again deemed minimal (Additional file 1: Table S3).

**Fig. 5:**
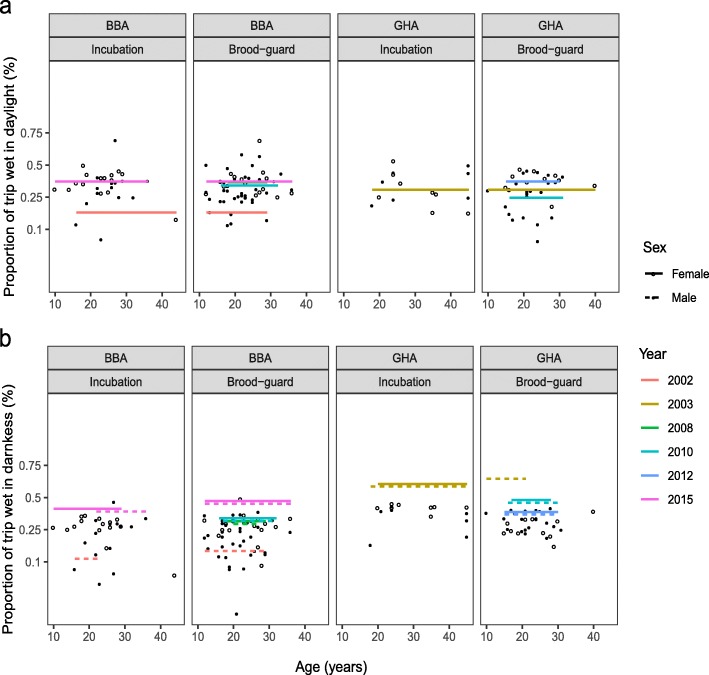
Relationship between age and low-resolution activity metrics for male (closed circles) and female (open circles) black-browed (BBA) and grey-headed (GHA) albatrosses during the incubation and brood-guard stages. Regression lines indicate the fitted values of the average of the most supported models for each response variable. Where a significant effect of sex was found, males (dotted) and females (solid) are shown with separate lines. Values of transformed response variables (**a** and **b**) are back-transformed on the y-axis but the scale of the transformation is retained

The overall proportion of the foraging trip spent wet during daylight and darkness varied between species and study year (Table 2, Fig. 5a and b). BBA spent on average 2% more of their trips wet during daylight than GHA, regardless of sex and breeding stage (Fig. 5a). The reverse was true in darkness, during which GHA spent 19% more of their trip on average on the water than BBA (Fig. 5b). This was apparent regardless of sex and breeding stage during daylight (Fig. 5a, Table 2). There was only weak evidence for an effect of these terms during darkness as they were not included in the top models as ranked by LOOCV (Additional file 1: Table S5). This activity metric fluctuated considerably between study years for BBA, especially during darkness (modelled 20 and 28% difference between the lowest and highest values in daylight and darkness, respectively). The variation among study years was less for GHA during daylight, but was comparable to that in BBA during darkness (modelled 12 and 25% difference between the lowest and highest values in daylight and darkness, respectively).

### Age-related variation in habitat preferences

**Fig. 6:**
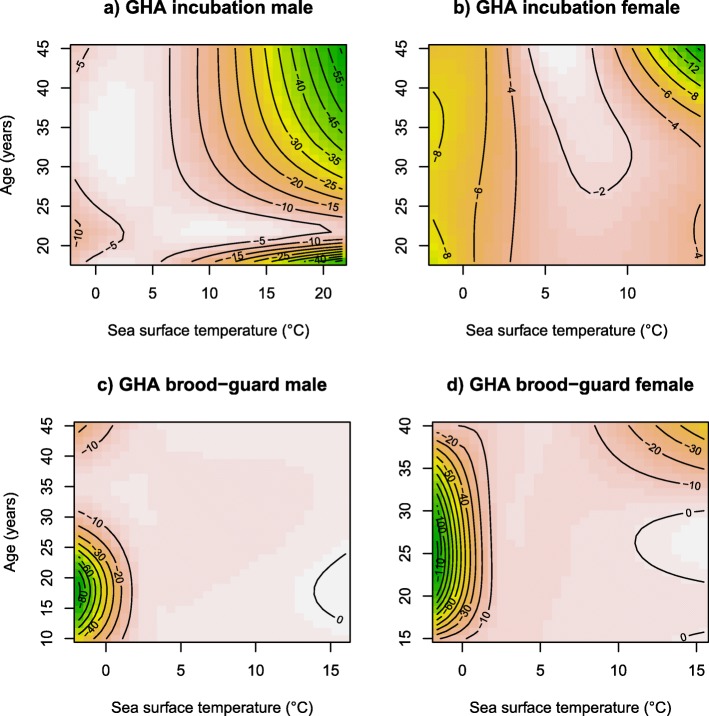
Contour plots (**a**-**d**) of most important variables explaining the distribution of grey-headed albatrosses (GHA) foraging bouts during the incubation and brood-guard breeding stages. Probability of foraging bout occurrence for birds of different ages and values of sea surface temperature is represented by color (high probability of occurrence; red, low probability of occurrence; green)

**Fig. 7:**
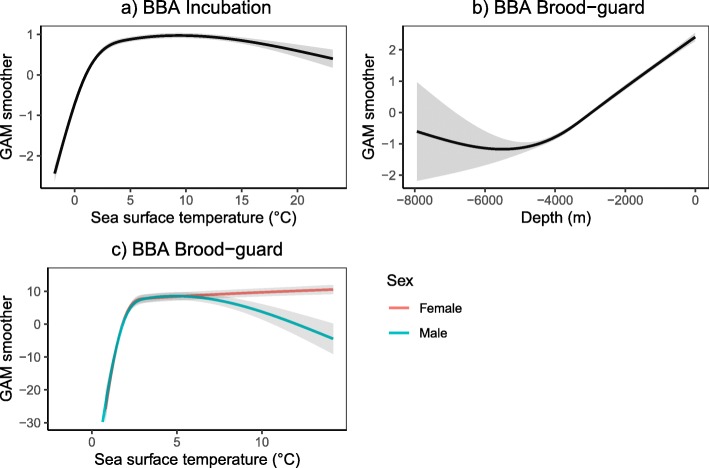
Response curves (**a**-**c**) of most important variables explaining the distribution of black-browed albatross (BBA) foraging bouts during the incubation and brood-guard breeding stages. Sex is represented by color for females (red) and males (blue) in plot C. Standard errors of the responses from model outputs are shown in grey

There was evidence for age-specific habitat preferences in the models predicting the distribution of foraging bouts of GHA but not BBA (Table 3, Figs. 6 and 7). The most important predictor of habitat use for GHA was ‘SST’ interacting with the ‘SEX’ and ‘AGE’ of the birds for both the incubation and brood-guard stages (Table 3, Fig. 6a-d). Excluding the youngest (18 years) incubating male GHA, which foraged in cold waters off the Antarctic Peninsula (Fig. 6a; 0–5 °C), model response contour plots indicated that during incubation, male and female GHA showed a progressive narrowing in temperature preference with increasing age (Fig. 6a and b). Indeed, younger birds of both sexes foraged indiscriminately across a wide range of SST (Fig. 6a and b; males: 2–20 °C, females: 3–14 °C), whereas older birds targeted specific habitats. Old males (40–45 years) avoided warmer waters to the north of South Georgia, preferentially foraging in colder southerly waters (Figs. 2 and 6a; 0–6 °C) and the oldest female (45 years) targeted an entirely separate foraging habitat to other females, to the north-west of the colony (Figs. 2 and 6b; 5–8 °C). During brood-guard, females similarly foraged within a narrowing temperature range with increasing age (Fig. 6d: 0–15 °C in 15–30 years and 0–10 °C in 35–40 years). This age-related shift in habitat preference was not as strong as in the incubation stage, presumably because movements and habitat choices were limited by the greater central-place constraint. In contrast, only young brooding male GHA showed a specific temperature preference, avoiding cold waters to the south of the colony (Figs. 2 and 6c; > 2 °C).

**Table 3:** Environmental predictor variables retained in the best models explaining the distribution of foraging bouts in black-browed albatrosses (BBA) and grey-headed albatrosses (GHA) during different breeding stages

Model predictors	DEPTH	DEPTH SD	SST	SST SD	CHL	WIND	SLA	EKE	AUC (sd)
Dataset									
BBA Incubation			x						0.76 ± 0.11
BBA Brood-guard	x		x, SST: Sex						0.89 ± 0.08
GHA Incubation			x, SST: Sex: Age						0.76 ± 0.12
GHA Brood-guard			x, SST: Sex: Age						0.81 ± 0.08

Habitat preference models were constructed separately for both species and for the incubation and brood-guard breeding stages. An ‘x’ indicates terms retained in the best model for each combination of species and breeding stage. Where an ‘x’ is followed by a colon and either ‘Sex’, ‘Age’ or ‘Sex: Age’ indicates a two or three-way interaction of those terms with that particular environmental predictor variable. Mean Area Under the Curve (AUC) scores and standard deviations (sd) of those scores for each model are indicated in the final column. Values of 0.5–0.7, 0.7–0.9 and > 0.9 represent poor, reasonable and very good model performance, respectively

The best models predicting the distribution of foraging bouts in GHA performed reasonably well, with AUC = 0.76 and AUC = 0.81 for the incubation and brood-guard stages respectively. However, the accuracy of the predictions when calculated separately for each individual varied more for the incubation stage, when the birds took longer trips, suggesting greater variability in their habitat preferences (AUC of 0.65–0.87) than during brooding (AUC of 0.73–0.89).

The most important predictor of habitat use in BBA during the incubation stage was also ‘SST’ but without any interaction with sex or age. Model response curves indicated that probability of foraging was highest in warmer waters between 4 and 15 °C (Fig. 7a) between the Antarctic Peninsula and Patagonian Shelf (Fig. 1a). ‘DEPTH’ was the most important predictor of habitat use of BBA during the brood-guard stage, followed by the interaction of ‘SST’ and ‘SEX’. Model response curves indicated that brooding BBA preferentially foraged in neritic waters close to the colony (Fig. 7b; the probability of foraging increased with decreasing depth). Female BBA preferentially foraged in waters spanning a wide range of temperatures (Figs. 1a and 7c; 2–15 °C) to the northwest of South Georgia, whereas males preferentially foraged in colder waters to the southwest (Figs. 1a and 7c; < 5 °C).

As with the models for GHA, the model of habitat preferences of BBA during brood-guard was more accurate than during incubation (AUC = 0.76 and AUC = 0.89, respectively), and varied less for BBA during incubation than brood-guard when calculated separately for each individual (AUC between 0.65 and 0.87, and between 0.71 and 0.97, respectively).

## Discussion

This study found evidence of extensive age-related variation in the foraging behavior of two congeneric, long-lived seabirds; black-browed (BBA) and grey-headed (GHA) albatrosses, during the breeding season. As we hypothesized, young breeders of both species displayed age-specific patterns in terms of trip duration (BBA and GHA), latitudinal distribution (BBA and GHA) and foraging activity at sea (BBA), but in contrast to our expectations, so did old breeders of both species. As predicted, effects of age were most apparent during incubation; however, there was evidence of age-specific activity patterns in BBA and habitat preferences in GHA irrespective of breeding stage, whereas older GHA segregated at-sea from younger birds during the brood-guard stage only. These findings highlight the complex interaction between the changing degree of the central-place constraint and the intrinsic attributes of individual seabirds in shaping foraging behavior.

### Age-related variation in foraging behavior in early adulthood

Naïve individuals show marked improvements in foraging performance during early life as they gain experience in how to move, navigate, locate prey and other skills [4, 89, 110]. Although many species of seabirds have a prolonged immaturity phase, individuals may require additional skills to forage successfully for both themselves and their young once they recruit into the breeding population [21, 43].

Here, the foraging behavior of young breeders of both albatross species differed initially from that of mid-age and old individuals (as seen in other species, [32, 72, 103]). Foraging trips were longer in young than mid-age BBA during the incubation stage, and they showed higher activity levels irrespective of breeding stage, landing more often and resting for less time on the water between landings. A previous tracking study at the Crozet Islands found that young (5-year-old) king penguins (*Aptenodytes patagonicus*) conducted longer trips than older individuals (9-year-olds), performed more dives (a proxy for foraging effort), and were less efficient at foraging [97]. As albatrosses are under strong selection to forage efficiently during the incubation stage to minimize the risk of their partner deserting before they return, our results suggest that reduced foraging competency contributes to the lower reproductive success observed in young BBA breeding at Bird Island [36]. It is difficult to verify this hypothesis without data on daily mass gain during trips or success rates of individual foraging bouts, but BBA recruit into the breeding population at a younger age than in other albatross species, and it seems likely they are still honing their skills in capturing, locating or handling prey [36, 103, 110]. Alternatively, BBA may need several breeding attempts to adapt to the new constraints imposed on foraging behavior by breeding, such as coordinating nest attendance with a mate, or competing for prey amongst high densities of conspecifics in waters around the colony [105]. Indeed, young BBA foraged at more southerly latitudes during the incubation stage but did not differ in habitat preferences from older birds, indicating they may avoid prey aggregations where competition is greatest, as seen in young wandering albatrosses (*Diomedea exulans*) [10].

Our analysis also suggested that young GHA took longer foraging trips than mid-age individuals during the incubation stage. This trend is to be interpreted with caution, however, as our sample of tracked birds was skewed towards older individuals ((all birds were ≥ 18 years old and GHA generally recruit at 13 years old), [36]). As young GHA during incubation also had wider habitat preferences than older birds in terms of sea surface temperature, the longer trips may have resulted from lower efficiency at locating profitable foraging habitats, as seen in young Cory’s shearwaters (*Calonectris borealis*) [43]. While it could be hypothesized that this behavior is representative of breeders in general (as the subset of tracked birds already had several years of breeding experience), our sample of brooding birds included very young breeders (10 years old was the minimum age), and these individuals had similarly wide habitat preferences. The increased severity of the central-place constraint during brood-guard did not constrain these preferences, and may explain lower breeding success in young GHA if they are unable to locate and deliver high-quality prey to their young [30, 56, 64].

Honing foraging skills over several breeding attempts may drive the within-individual improvement in breeding success observed in early adulthood in BBA and GHA [36], which could be tested by longitudinal tracking studies of individuals over several years. Alternatively, there may be selection for high-quality individuals with specific foraging strategies (short trip durations, low landing rate, more northerly distributions, [31, 65]), or poor environmental conditions (via food scarcity) may disproportionally affect the foraging success of naïve individuals in certain years [43].

### Age-related variation in foraging behavior in late adulthood

In late adulthood, in contrast to our expectations, GHA as well as BBA showed signs of age-related changes in foraging behavior, even in the absence of significant population-level reproductive senescence in BBA [36]. Furthermore, the changes in certain foraging traits occurred at a later age than recorded population-level declines in breeding success, while other changes occurred progressively with age, suggesting there is a complex relationship between foraging and reproductive performance in these two species [36].

Foraging trip duration in incubation increased in GHA from age 26 onwards. This confirms the results of a previous study at Bird Island in the 2002/03 breeding season which found that old (≥ 35 years old) males took longer trips than mid-aged (≤28 years old) males [19]. These older male GHA also showed reduced foraging and breeding performance, suggesting they may be constrained by some degree of physical deterioration in old age [28]. Benefiting from a larger dataset, we also found that female GHA took longer trips with increasing age. Differences between age groups in performance might only be apparent when conditions are sub-optimal, and it could be hypothesized that females encountered particularly unfavorable conditions at sea in 2011/12 compared to 2002/03 [94]. Older incubating birds of both sexes also showed a progressive change in preferred foraging habitat with increasing age in that they targeted a narrowing range of sea surface temperatures. This pattern could indicate a further increase in foraging efficiency with age, with birds targeting predictably productive areas learned through experience [43]. However, old incubating GHA did not forage within areas particularly rich in their preferred prey (the squid, *Martialia hyadesi*) [112] and habitat selection in old individuals of a number of taxa is mediated by age-related increases in the incidence of disease or injury [44, 46, 62]. Indeed, it has been suggested that senescent female Soay sheep (*Ovis aries*) have smaller home ranges of lower quality as a result of competitive exclusion by younger conspecifics, and that male wandering albatrosses forage progressively further south with increasing age to reduce foraging costs by flying in windier areas [35, 44, 52] . These two theories may explain the behavior seen in GHA in our study, especially as the oldest birds foraged in more southerly and windier areas during the brood-guard stage.

Increased foraging trip duration in older BBA also suggests they experience senescence in foraging performance, as hypothesized for GHA. BBA do not show reproductive senescence, however, and hence they may be able to maintain high foraging efficiency in spite of potential physiological decline. Similarly, old Brünnich’s guillemots (*Uria lomvia*) did not differ in dive behavior from young birds, but had lower blood oxygen stores, resting metabolic rate and thyroid hormone levels [33]. In accordance with the so-called ‘restraint’ hypothesis [108], taking longer trips may be an energy-saving tactic, which would allow BBA to offset physiological deterioration, and maintain a consistent level of foraging efficiency and hence reproductive success into old age. BBA also showed a progressive decrease in foraging effort with increasing age, landing less often and resting for longer on the water between landings, which may reflect this energy-saving tactic. Indeed, while this trend could imply that old birds are simply more efficient at foraging, old (20+ years) wandering albatrosses tracked from Bird island during the non-breeding season that landed more often on the water were less likely to breed successfully the following year [21]. The study that investigated reproductive aging on Bird Island included few BBA older than 40 years of age [36], and it is possible that the change we observed in foraging behavior in old age eventually affects average reproductive success, but only in very old birds.

It is noteworthy that progressively longer foraging trips during incubation were apparent from the same point in late adulthood in both species, even though BBA are annual breeders and hence senescence should in theory commence earlier and develop more quickly than in GHA, which breed biennially [47, 96]. Further research may reveal whether this difference indicates a true deviation from life-history theory or is unrelated to breeding success. BBA taking shorter trips may have been exposed to high incidental mortality in fisheries operating historically around South Georgia, resulting in the selective disappearance of birds that take shorter foraging trips [29]. Alternatively, there may be an effect of the environment experienced by these birds on their aging trajectories, considering that BBA and GHA forage largely in different areas during breeding, overlap very little at sea during the nonbreeding season and were tracked in separate years [78, 84]. Environmental effects may also explain why wandering albatrosses breeding at Bird Island showed no obvious changes in foraging behavior with age in spite of age-related variation in breeding success [35].

### Other drivers of foraging behavior during the breeding season

Within species, the intensity of aging often varies according to sex, in association with the strength of sexual selection, and the cost of producing or maintaining sexually selected traits or behaviors [2, 23, 59]. We found no strong evidence for an interaction between the sex and age of individual GHA and BBA on their foraging behavior, despite the sexual dimorphism in wing area and wing loading in both species, and the higher chick provisioning rate of male BBA [45, 78]. However, females of both species did make longer foraging trips during both breeding stages, and female BBA rested for longer on the water between foraging bouts than males during daylight. These trends suggest that females of both species allocate more effort to self-maintenance, as seen for example in female little auks (*Alle alle)* which take long self-feeding trips to replenish body reserves used during egg production [107]. This behavior may enable females of both species to achieve a longer reproductive lifespan, whereas males may pay a physiological price for maintaining higher levels of foraging effort [16, 36]. Otherwise, females of both GHA and BBA foraged at more northerly latitudes than males during incubation, in keeping with previous research which attributed this spatial segregation to differences in flight performance [78].

BBA showed no age-specific habitat preferences, but instead preferentially foraged within a wide range of relatively warm sea surface temperatures during both breeding stages. Probability of foraging with respect to SST peaked at around 3 °C and remained constant thereafter in females, but decreased in males in waters above 5 °C during the brood-guard stage. This difference in preference may indicate that male and female BBA have differing nutritional demands that induce them to target prey that associate with particular temperature regimes (as suggested for northern gannets (*Morus bassanus*), [55]). Alternatively, it may relate to the more northerly distribution of female BBA during brood-guard for other reasons (e. g. related to wind regime preferences, [78]). Both sexes also preferentially foraged in shallow waters, most likely as they were constrained to remain close to the colony during this breeding stage [85]. Our analyses did not find preferences for quite the same suite of environmental covariates that predicted habitat use in previous studies of both BBA and GHA, for instance eddy kinetic energy or chlorophyll concentration [90, 99]. However, our sample differed from those studies in that it only included birds of known age and sex, and there is always considerable individual and annual variability in preferred foraging habitats [76, 111].

There were no obvious age-specific patterns in terms of activity budgets. BBA spent a larger proportion of time on the water during the day, and a smaller proportion on the water at night than GHA. These findings match previous research suggesting a degree of specialization in feeding behavior between these two species, perhaps as a result of competition [74]. In addition, activity metrics, as well as maximum foraging range, varied between years in both species indicating these birds show flexibility in response to varying environmental conditions and, consequently, distribution or availability of prey. This differs from previous research suggesting that the smaller albatrosses (*Thalassarche* and *Phoebetria* species) have similar overall energy budgets [104]. Finally, additional fine-scale activity data is needed for GHA of known age, as there may be age-specific changes that we were unable to detect.

## Conclusion

Here we demonstrated that several aspects of the foraging behavior of black-browed and grey-headed albatrosses breeding at South Georgia were related to age. While this study was purely cross-sectional, and inferences about the consequences of foraging behavior for fitness could not be tested at the individual level, it nevertheless identified some clear patterns at the population-level. As more studies seek to better link tracking data to physiology and life-history decisions and events of individuals, there will be increasing opportunity to ask complex questions regarding relationships between age-specific variation in behavioral traits and multiple aspects of fitness ((breeding success, timing of breeding, chick growth rates etc.), [26]). These questions are of fundamental ecological and evolutionary interest [86] and are likely to have important consequences for the population dynamics of these threatened albatrosses as well as other species of long-lived seabirds [17, 27, 75]. Young or old individuals may be disproportionally impacted by poor environmental conditions because of lower foraging efficiency or differences in distribution, and such changes are likely to become more prevalent under predicted scenarios of global warming [94]. Marine protection measures could benefit some age and sex classes more than others, and potentially target young and mid-aged individuals that will make the most contribution to population growth rate over the long term [63].

## Supplementary information

**Additional file 1.** Supplementary information on datasets (sample sizes) and methods (labelling foraging bouts, determining number of pseudo-absences, model selection tables and parameter estimates).

## Data Availability

The datasets supporting the conclusions of this article are available for download from the BirdLife International Seabird Tracking Database. (http://seabirdtracking.org/mapper/contributor.php?contributor_xml:id=361; dataset ids: 457, 459, 492 and 494).

## Abbreviations

AIC: Akaike information criterion
AICc: Akaike information criterion adjusted for small sample sizes
AUC: Area under the curve
BBA: Black-browed albatross
CHL: Chlorophyll
EKE: Eddy kinetic energy
GHA: Grey-headed albatross
GPS: Global positioning system
PTT: Platform terminal transmitter
SST: Sea surface temperature
