# Supplementary material for: Effects of age on foraging behavior in two closely related albatross species
Source: Mov Ecol. 2020 Feb 7;8:7. doi: 10.1186/s40462-020-0194-0 (PMC7006180; doi:10.1186/s40462-020-0194-0)
Supplement: Supplementary file 1 — Additional file 1. Supplementary information on datasets (sample sizes) and methods (labelling foraging bouts, determining number of pseudo-absences, model selection tables and parameter estimates). [file 40462_2020_194_MOESM1_ESM.docx]

**Additional file 1**

**Table S1. Available sample sizes of processed location and immersion data of black-browed (BBA) and grey-headed (GHA) albatrosses birds of known sex and breeding stage. The sampling interval indicates the setting used for the GPS loggers, or average fix interval for the Platform Terminal Transmitters (PTTs). Trips from birds of known age or of an estimated minimum age greater than the age at which each species shows reproductive senescence (Froy et al. 2017) are in blue font. INC – Incubation, BR – brood-guard**

| Species | Stage | Season | Device | Sampling interval (minutes) | No. birds | No. trips | Age range | Sex ratio (F:M) | Trips with immersion data | |
| --- | --- | --- | --- | --- | --- | --- | --- | --- | --- | --- |
|  | | | | | | | | | High-res | Lower-res |
| BBA | INC | 1997 | PTT | 224 | 10 (4) | 10 (4) | 14 – 24 | 1 : 3 |  |  |
|  |  | 2002 | PTT | 83 | 20 (7) | 20 (7) | 10 – 44 | 1 : 6 |  | 15 (6) |
|  |  | 2015 | GPS | 30 | 37 (28) | 37 (28) | 10 – 36 | 15 : 13 | 33 (27) |  |
|  | BR | 1994 | PTT | 144 | 3 | 4 |  |  |  |  |
|  |  | 2002 | PTT | 65 | 22 (9) | 22 (9) | 12 – 29 | 0 : 9 |  | 22 (9) |
|  |  | 2005 | PTT | 116 | 3 | 4 |  |  |  |  |
|  |  | 2008 | GPS | 5 | 18 (12) | 18 (12) | 18 – 29 | 4 : 8 | 21 (11) |  |
|  |  | 2010 | GPS | 10 | 32 (29) | 33 (28) | 14 – 32 | 11 : 17 | 12 (10) | 16(16) |
|  |  | 2015 | GPS | 10 | 28 (19) | 63 (49) | 12 – 36 | 6 : 16 | 63 (49) |  |
| GHA | INC | 1996 | PTT | 133 | 3 (1) | 3 (1) | 38 | 0 : 1 |  |  |
|  |  | 2003 | GPS | 60 | 3 (1) | 3 (1) | 18 | 1 : 0 |  |  |
|  |  | 2003 | PTT | 57 | 28 (17) | 28 (17) | 18 – 45 | 8 : 9 |  | 20 (16) |
|  | BR | 1993 | PTT | 195 | 1 | 1 |  |  |  |  |
|  |  | 1995 | PTT | 133 | 6 | 3 |  |  |  |  |
|  |  | 2003 | PTT | 54 | 28 (7) | 17 (12) | 10 – 45 | 2 : 5 |  | 27 (11) |
|  |  | 2010 | GPS | 30 | 26 (17) | 29 (20) | 10 – 45 | 3 : 14 | 9 (6) | 15 (14) |
|  |  | 2012 | GPS | 10 | 23 (14) | 25 (14) | 15 – 30 | 8 : 6 |  | 27 (27) |


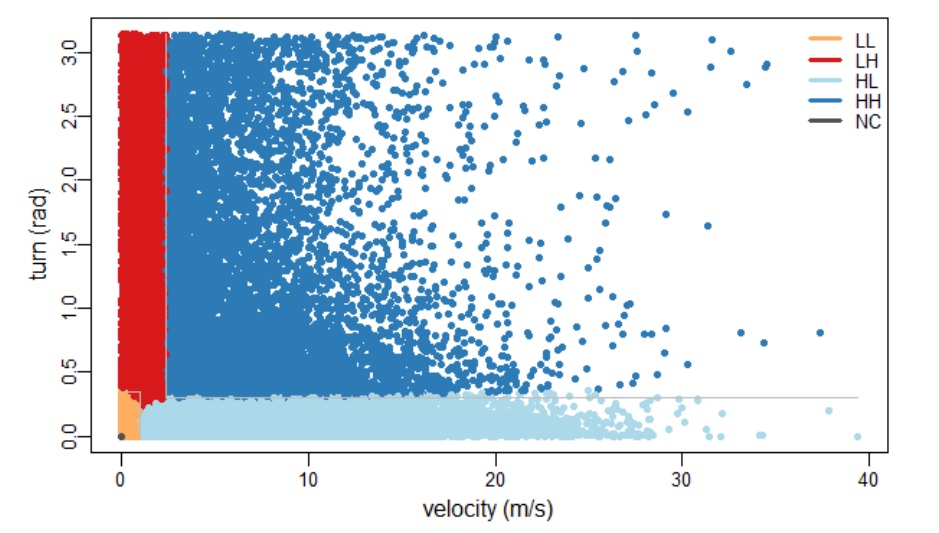


**Figure S1. Clustering scatterplot of trip locations according to states identified using EMbC. ‘L’ and ‘H’ indicate low and high values of local turning angle and velocity values. Behaviours LH and HH were subsequently merged. NC represents the first point of each trip, which the algorithm did not classify, re-labelled as behaviour HL.**

**Table S2. Verification of the EMbC population-level analysis using information on the (1) proportion of all trips classified as states 1-3, (2) proportion of total landings occurring in each state, (3) proportion of time spent wet in each state, and (4) mean landing rate (wet events per hour) during each state. This table summarises the characteristics of 74 trips with corresponding immersion data.**

| States | Behaviour | Prop. trips | Prop. total landings | Mean prop. wet | Mean landing rate |
| --- | --- | --- | --- | --- | --- |
| 1 | Resting | 0.17 | 0.18 | 0.69 | 0.27 |
| 2 | Foraging | 0.40 | 0.50 | 0.39 | 0.32 |
| 3 | Transit | 0.43 | 0.33 | 0.23 | 0.20 |


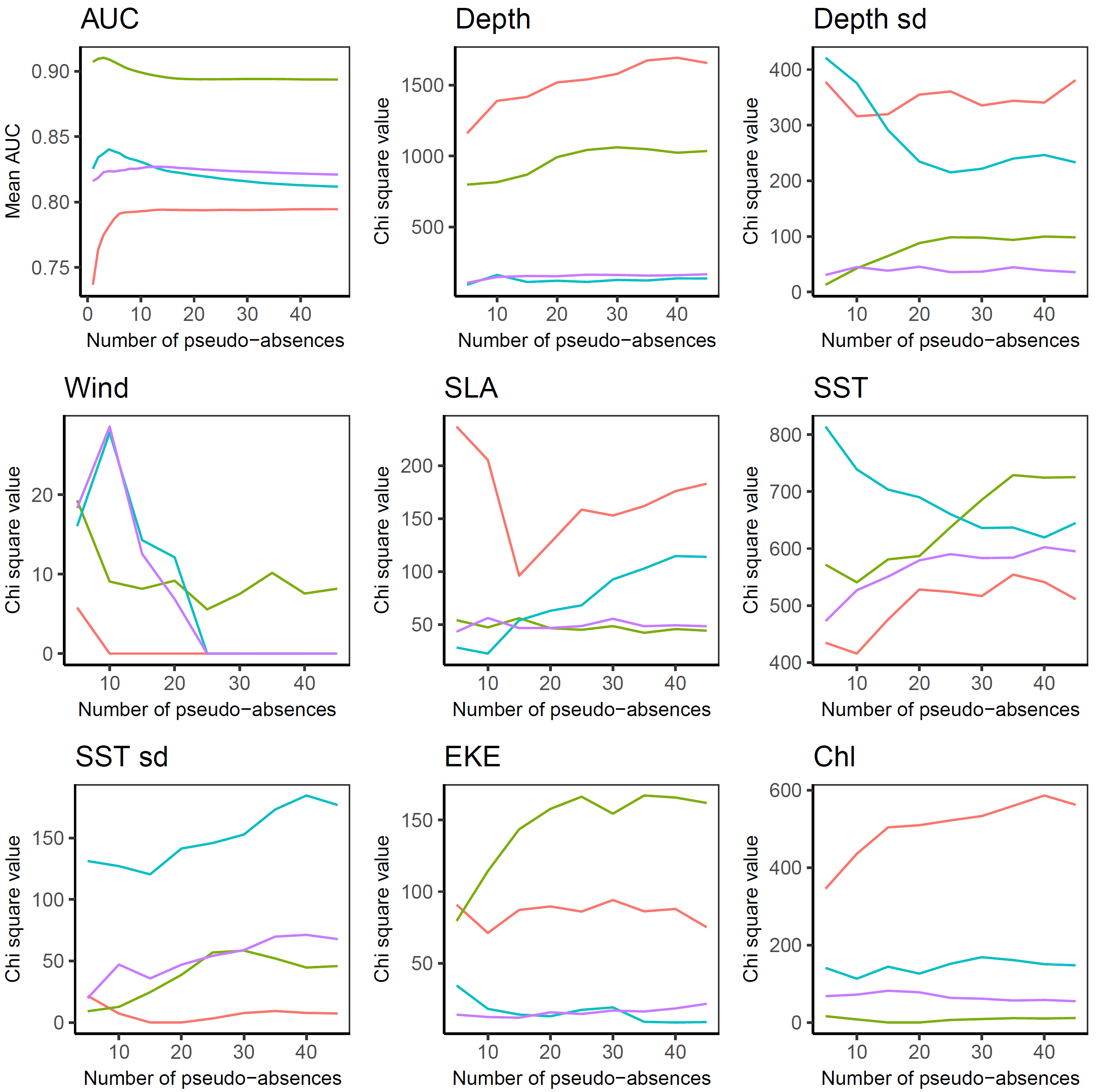

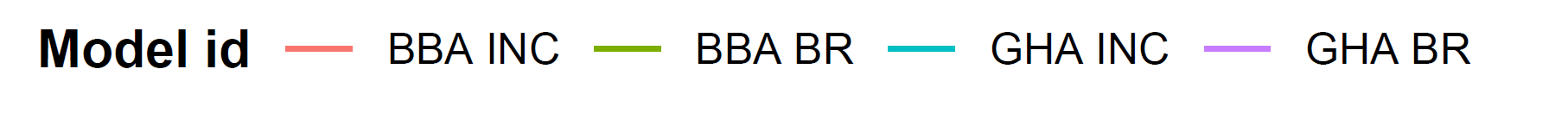


**Figure S2. Mean Area Under the Curve (AUC; first plot) and Chi square value (all other plots) of environmental predictor variables for an increasing number of pseudo-absences (between 1 and 47) for the four models predicting the habitat preferences of black-browed (BBA) and grey-headed (GHA) albatrosses in the incubation (INC) and brood-guard (BR) stages. ‘Depth’ is bathymetry, ‘Depth sd’ is bathymetric slope, ‘Wind’ is wind speed, ‘SLA’ is sea level anomaly, ‘SST’ is sea surface temperature, ‘SST sd’ is the standard deviation of SST, ‘EKE’ is eddy kinetic energy and ‘Chl’ is chlorophyll concentration.**

**Table S3. Effect of age, sex, stage, species and year on trip characteristics and activity patterns of black-browed and grey-headed breeding at Bird Island, South Georgia** **. ‘x’ indicates terms retained in the most supported models for each response variable (< 2Δ AICc of the top model)**. AICc= Akaike information criterion model score; ΔAICc = difference in Akaike information criterion score between models; w=Akaike information criterion weights calculated for the set of most supported models.

|  |  | **Predictor variables** | | | | | | | | | | | | | | |  |  |  |  |
| --- | --- | --- | --- | --- | --- | --- | --- | --- | --- | --- | --- | --- | --- | --- | --- | --- | --- | --- | --- | --- |
| **Response variable** | **n** | Age | Age^2^ | Sex | Stage | Species | Year | Age:Sex | Age^2^:  Sex | Age:  Stage | Age^2^:  Stage | Age:  Species | Age^2^:  Species | Sex:  Stage | Sex:  Species | Stage:  Species | **df** | **AICc** | **ΔAICc** | **w** |
| Trip duration (days) | 158 | x | x | x | x | x |  |  |  | x | x | x |  |  |  |  | 10 | 213.5 | 0.000 | 0.569 |
|  |  | x | x | x | x | x |  |  |  | x | x |  |  |  |  |  | 9 | 214.1 | 0.553 | 0.431 |
| Max range from colony (km) | 158 |  |  | x | x | x | x |  |  |  |  |  |  |  | x |  | 12 | 338.69 | 0.000 | 1.000 |
| Latitude at max range (°) | 158 | x | x | x | x | x |  |  |  | x | x | x |  | x |  | x | 12 | 874.2 | 0.000 | 0.448 |
|  |  | x | x | x | x | x |  |  |  | x |  | x |  | x |  | x | 11 | 874.9 | 0.688 | 0.317 |
|  |  | x | x | x | x | x |  |  |  | x | x |  |  | x |  | x | 11 | 875.5 | 1.287 | 0.235 |
| Landings.hr^-1^ in daylight^a^ | 66 | x |  | x |  |  | x |  |  |  |  |  |  |  |  |  | 6 | 69.76 | 0.000 | 0.535 |
|  |  | x |  |  |  |  | x |  |  |  |  |  |  |  |  |  | 5 | 71.42 | 1.658 | 0.233 |
|  |  | x |  | x |  |  |  | x |  |  |  |  |  |  |  |  | 5 | 71.43 | 1.674 | 0.232 |
| Landings.hr^-1^ in darkness^a^ | 64 |  |  |  |  |  | x |  |  |  |  |  |  |  |  |  | 4 | 116.0 | 0.000 | 1.000 |

Table S3 continued

|  |  | **Predictor variables** | | | | | | | | | | | | | | |  |  |  |  |
| --- | --- | --- | --- | --- | --- | --- | --- | --- | --- | --- | --- | --- | --- | --- | --- | --- | --- | --- | --- | --- |
| **Response variable** | **n** | Age | Age^2^ | Sex | Stage | Species | Year | Age:Sex | Age^2^:  Sex | Age:  Stage | Age^2^:  Stage | Age:  Species | Age^2^:  Species | Sex:  Stage | Sex:  Species | Stage:  Species | **df** | **AICc** | **ΔAICc** | **w** |
| Wet bout length in daylight (mins)^a^ | 66 | x |  | x |  |  |  |  |  |  |  |  |  |  |  |  | 4 | 97.77 | 0.000 | 1.000 |
| Wet bout length in darkness (mins)^a^ | 64 |  |  | x |  |  | x |  |  |  |  |  |  |  |  |  | 5 | 144.8 | 0.000 | 0.523 |
|  |  |  |  |  |  |  | x |  |  |  |  |  |  |  |  |  | 4 | 146.1 | 1.238 | 0.282 |
|  |  | x |  | x | x |  |  |  |  |  |  |  |  | x |  |  | 6 | 146.8 | 1.978 | 0.195 |
| Prop daylight wet (%) | 130 |  |  |  |  | x | x |  |  |  |  |  |  |  |  |  | 8 | 199.3 | 0.000 | 1.000 |
| Prop darkness wet (%) | 128 |  |  | x | x | x | x |  |  |  |  |  |  |  |  |  | 10 | 314.8 | 0.000 | 0.386 |
|  |  |  |  |  | x | x | x |  |  |  |  |  |  |  |  |  | 9 | 315.1 | 0.325 | 0.328 |
|  |  |  |  |  |  | x | x |  |  |  |  |  |  |  |  |  | 8 | 315.4 | 0.608 | 0.285 |

^a^ Species was not included in the model for these two metrics as sample size was very small for GHA.

**Table S4. Parameter estimates and standard errors (SE) for the best-supported models, predicting the trip characteristics and activity patterns of black-browed and grey-headed albatrosses breeding at Bird Island, South Georgia as presented in Table 2. Parameter estimates and SEs are also shown for the average of the best-supported-models (if >1 best-supported model) as presented in Figures 3-5.**

|  |  | **Estimate ± SE** | | | |
| --- | --- | --- | --- | --- | --- |
| **Variable** | **n** | **Model 1** | **Model 2** | **Model 3** | **Model average** |
| ***sqrt (Trip duration)*** | 158 |  |  |  |  |
| Intercept |  | 4.760 ± 0.545 | 4.604 ± 0.540 |  | 4.693 ± 0.548 |
| Age |  | -0.159 ± 0.041 | -0.140 ± 0.039 |  | -0.151 ± 0.041 |
| Age^2^ |  | 0.003 ± 0.001 | 0.003 ± 0.001 |  | 0.003 ± 0.001 |
| Sex (Female) |  | 0.205 ± 0.077 | 0.207 ± 0.078 |  | 0.206 ± 0.077 |
| Species (GHA) |  | 0.701 ± 0.293 | 0.238 ± 0.080 |  | 0.501 ± 0.323 |
| Stage (Brood-guard) |  | -3.018 ± 0.709 | -2.866 ± 0.707 |  | -2.953 ± 0.712 |
| Age : Species (GHA) |  | -0.019 ± 0.012 | - |  | -0.011 ± 0.013 |
| Age : Stage (Brood-guard) |  | 0.126 ± 0.055 | 0.115 ± 0.054 |  | 0.121 ± 0.055 |
| Age^2^: Stage (Brood-guard) |  | -0.002 ± 0.001 | -0.002 ± 0.001 |  | -0.002 ± 0.001 |
|  |  |  |  |  |  |
| ***log(Maximum range)*** | 158 |  |  |  |  |
| Intercept |  | 6.456 ± 0.339 |  |  |  |
| Year (2002) |  | 0.361 ± 0.395 |  |  |  |
| Year (2003) |  | -0.217 ± 0.429 |  |  |  |
| Year (2008) |  | -0.130 ± 0.420 |  |  |  |
| Year (2010) |  | 0.095 ± 0.395 |  |  |  |
| Year (2012) |  | 0.328 ± 0.473 |  |  |  |
| Year (2015) |  | -0.401 ± 0.360 |  |  |  |
| Sex (Female) |  | 0.824 ± 0.146 |  |  |  |
| Species (GHA) |  | 0.879 ± 0.218 |  |  |  |
| Stage (Brood-guard) |  | -1.053 ± 0.155 |  |  |  |
| Sex (Female) : Species (GHA) |  | -0.838 ± 0.244 |  |  |  |
|  |  |  |  |  |  |
| ***Latitude at maximum range*** | 158 |  |  |  |  |
| Intercept |  | -69.554 ± 4.426 | -64.910 ± 3.488 | -70.902 ± 4.401 | -68.398 ± 4.807 |

Table S4 continued

|  |  | **Estimate ± SE** | | | |
| --- | --- | --- | --- | --- | --- |
| **Variable** | **n** | **Model 1** | **Model 2** | **Model 3** | **Model average** |
| Age |  | 1.107 ± 0.328 | 0.742 ± 0.249 | 1.281 ± 0.317 | 1.032 ± 0.368 |
| Age^2^ |  | -0.015 ± 0.006 | -0.008 ± 0.005 | -0.01 ± 0.005 | -0.014 ± 0.007 |
| Sex (Female) |  | 7.726 ± 1.041 | 7.682 ± 1.047 | 7.716 ± 1.049 | 7.710 ± 1.045 |
| Species (GHA) |  | 2.128 ± 2.802 | 2.466 ± 2.813 | -2.535 ± 1.231 | 1.139 ± 3.248 |
| Stage (Brood-guard) |  | 13.978 ± 5.746 | 5.107 ± 2.332 | 15.210 ± 5.753 | 11.453 ± 6.575 |
| Age : Species (GHA) |  | -0.180 ± 0.097 | -0.199 ± 0.097 | - | -0.143 ± 0.117 |
| Age : Stage (Brood-guard) |  | -1.006 ± 0.438 | -0.283 ± 0.091 | -1.104 ± 0.438 | -0.799 ± 0.509 |
| Age^2^ : Stage (Brood-guard) |  | 0.014 ± 0.008 | - | 0.015 ± 0.008 | 0.010 ± 0.009 |
| Sex (Female) : Stage (Brood-guard) |  | -5.264 ± 1.296 | -5.218 ± 1.304 | -5.201 ± 1.306 | -5.235 ± 1.301 |
| Species (GHA) : Stage (Brood-guard) |  | 5.452 ± 1.458 | 5.581 ± 1.465 | 5.984 ± 1.441 | 5.618 ± 1.472 |
|  |  |  |  |  |  |
| ***log(Landing rate during daylight)*** | 66 |  |  |  |  |
| Intercept |  | 1.436 ± 0.229 | 1.316 ± 0.225 | 1.219 ± 0.269 | 1.358 ± 0.254 |
| Age |  | -0.020 ± 0.008 | -0.017 ± 0.008 | -0.007 ± 0.010 | -0.016 ± 0.010 |
| Year (2010) |  | -0.161 ± 0.174 | -0.173 ± 0.178 | - | -0.126 ± 0.168 |
| Year (2015) |  | 0.179 ± 0.130 | 0.165 ± 0.132 | - | 0.135 ± 0.136 |
| Sex (Female) |  | -0.192 ± 0.097 | - | 0.449 ± 0.398 | 0.002 ± 0.329 |
| Age : Sex (Female) |  | - | - | -0.026 ± 0.016 | -0.006 ± 0.014 |
|  |  |  |  |  |  |
| ***log(Landing rate during darkness)*** | **64** |  |  |  |  |
| Intercept |  | 0.439 ± 0.173 |  |  |  |
| Year (2010) |  | 0.676 ± 0.258 |  |  |  |
| Year (2015) |  | 0.275 ± 0.193 |  |  |  |
|  |  |  |  |  |  |
| ***log(Wet bout duration in daylight)*** | **66** |  |  |  |  |
| Intercept |  | 0.956 ± 0.257 |  |  |  |
| Age |  | 0.030 ± 0.010 |  |  |  |
| Sex |  | 0.269 ± 0.122 |  |  |  |
|  |  |  |  |  |  |
| ***log(Wet bout duration in darkness)*** | **64** |  |  |  |  |
| Intercept |  | 2.742 ± 0.224 | 2.864 ± 0.219 | 0.150 ± 0.463 | 2.535 ± 0.585 |
| Year (2010) |  | -0.803 ± 0.320 | -0.776 ± 0.326 | - | -0.639 ± 0.427 |
| Year (2015) |  | -0.235 ± 0.240 | -0.205 ± 0.244 | - | -0.181 ± 0.235 |

Table S4 continued

|  |  | **Estimate ± SE** | | | |
| --- | --- | --- | --- | --- | --- |
| **Variable** | **n** | **Model 1** | **Model 2** | **Model 3** | **Model average** |
| Sex (Female) |  | 0.334 ± 0.180 | - | 0.912 ± 0.300 | 0.353 ± 0.361 |
| Age |  | - | - | 0.027 ± 0.015 | 0.005 ± 0.013 |
| Stage (Brood-guard) |  | - | - | 0.428 ± 0.262 | 0.083 ± 0.205 |
| Sex (Female) : Stage (Brood-guard) |  | - | - | -0.927 ± 0.388 | -0.180 ± 0.405 |
|  |  |  |  |  |  |
| ***logit(Prop daylight wet)*** | **130** |  |  |  |  |
| Intercept |  | -1.624 ± 0.139 |  |  |  |
| Year (2003) |  | 1.197 ± 0.245 |  |  |  |
| Year (2008) |  | 1.041 ± 0.205 |  |  |  |
| Year (2010) |  | 0.927 ± 0.174 |  |  |  |
| Year (2012) |  | 1.482 ± 0.258 |  |  |  |
| Year (2015) |  | 1.060 ± 0.162 |  |  |  |
| Species (GHA) |  | -0.419 ± 0.166 |  |  |  |
|  |  |  |  |  |  |
| ***logit(Prop darkness wet)*** | **128** |  |  |  |  |
| Intercept |  | -2.192 ± 0.269 | -2.142 ± 0.269 | -1.899 ± 0.221 | -2.092 ± 0.285 |
| Year (2003) |  | 1.824 ± 0.406 | 1.933 ± 0.403 | 1.764 ± 0.391 | 1.843 ± 0.406 |
| Year (2008) |  | 0.842 ± 0.334 | 0.925 ± 0.332 | 1.033 ± 0.327 | 0.924 ± 0.340 |
| Year (2010) |  | 0.946 ± 0.286 | 1.025 ± 0.284 | 1.133 ± 0.277 | 1.025 ± 0.293 |
| Year (2012) |  | 0.489 ± 0.423 | 0.649 ± 0.413 | 0.757 ± 0.410 | 0.618 ± 0.431 |
| Year (2015) |  | 1.602 ± 0.280 | 1.691 ± 0.276 | 1.541 ± 0.260 | 1.614 ± 0.279 |
| Sex (Female) |  | 0.240 ± 0.151 | - | - | 0.093 ± 0.150 |
| Species (GHA) |  | 0.646 ± 0.263 | 0.610 ± 0.263 | 0.610 ± 0.265 | 0.624 ± 0.264 |
| Stage (Brood-guard) |  | 0.396 ± 0.224 | 0.351 ± 0.224 | - | 0.268 ± 0.255 |

**Table S5. Effect of age, sex, stage, species and year on trip characteristics and activity patterns of black-browed and grey-headed breeding at Bird Island, South Georgia.** ‘x’ indicates terms retained in the most supported models (top 5 are shown here) ranked according to Leave One Out Cross Validation (Loocv). AICc values are shown for comparison, and the most supported models chosen according to AICc are highlighted in blue for each response variable.

|  |  | **Predictor variables** | | | | | | | | | | | | | | |  |  |  |
| --- | --- | --- | --- | --- | --- | --- | --- | --- | --- | --- | --- | --- | --- | --- | --- | --- | --- | --- | --- |
| **Response variable** | **n** | Age | Age^2^ | Sex | Stage | Species | Year | Age:Sex | Age^2^:  Sex | Age:  Stage | Age^2^:  Stage | Age:  Species | Age^2^:  Species | Sex:  Stage | Sex:  Species | Stage:  Species | **df** | **Loocv** | **AICc** |
| Trip duration (days) | 158 | x | x | x | x | x |  |  |  | x | x | x |  |  |  |  | 10 | 0.473 | 213.5 |
|  |  | x | x | x | x | x |  |  |  | x | x |  |  |  |  |  | 9 | 0.476 | 214.1 |
|  |  | x | x | x | x | x |  |  |  |  |  |  |  |  |  |  | 7 | 0.477 | 215.1 |
|  |  | x | x |  | x | x |  |  |  | x | x | x |  |  |  |  | 9 | 0.483 | 218.5 |
|  |  | x | x | x | x |  |  |  |  | x | x |  |  |  |  |  | 8 | 0.484 | 221.0 |
| Max range from colony (km) | 158 |  |  | x | x | x | x |  |  |  |  |  |  |  | x | x | 13 | 0.697 | 339.0 |
|  |  |  |  | x | x | x | x |  |  |  |  |  |  |  | x |  | 12 | 0.698 | 338.7 |
|  |  |  |  | x | x | x |  |  |  |  |  |  |  |  | x | x | 7 | 0.708 | 344.1 |
|  |  |  |  | x | x | x |  |  |  |  |  |  |  |  | x |  | 6 | 0.712 | 344.7 |
|  |  |  |  | x | x | x | x |  |  |  |  |  |  |  |  | x | 12 | 0.716 | 346.8 |
| Latitude at max range (°) | 158 | x | x | x | x | x |  |  |  | x | x | x |  | x |  | x | 12 | 3.888 | 874.2 |
|  |  | x | x | x | x | x |  |  |  | x | x |  |  | x |  | x | 11 | 3.890 | 875.5 |
|  |  | x | x | x | x | x |  |  |  | x |  | x |  | x |  | x | 11 | 3.911 | 874.9 |

Table S5 continued

|  |  | **Predictor variables** | | | | | | | | | | | | | | |  |  |  |
| --- | --- | --- | --- | --- | --- | --- | --- | --- | --- | --- | --- | --- | --- | --- | --- | --- | --- | --- | --- |
| **Response variable** | **n** | Age | Age^2^ | Sex | Stage | Species | Year | Age:Sex | Age^2^:  Sex | Age:  Stage | Age^2^:  Stage | Age:  Species | Age^2^:  Species | Sex:  Stage | Sex:  Species | Stage:  Species | **df** | **Loocv** | **AICc** |
| Latitude at max range (°) - Continued | 158 | x |  | x | x | x |  |  |  |  |  | x |  | x |  | x | 10 | 3.913 | 876.2 |
|  |  | x | x | x | x | x |  |  |  | x |  |  |  | x |  | x | 10 | 3.927 | 877.0 |
| Landings.hr^-1^ in daylight* | 66 | x |  | x |  |  | x |  |  |  |  |  |  |  |  |  | 6 | 0.404 | 69.76 |
|  |  | x |  | x |  |  |  | x |  |  |  |  |  |  |  |  | 5 | 0.407 | 71.43 |
|  |  | x |  | x |  |  |  |  |  |  |  |  |  |  |  |  | 4 | 0.410 | 71.92 |
|  |  | x |  |  |  |  | x |  |  |  |  |  |  |  |  |  | 5 | 0.411 | 71.42 |
|  |  | x |  |  |  |  |  |  |  |  |  |  |  |  |  |  | 3 | 0.413 | 73.11 |
| Landings.hr^-1^ in darkness* | 64 |  |  |  |  |  | x |  |  |  |  |  |  |  |  |  | 4 | 0.589 | 116.0 |
|  |  |  |  |  |  |  |  |  |  |  |  |  |  |  |  |  | 2 | 0.600 | 118.4 |
| Wet bout duration in daylight (mins)* | 66 | x |  | x |  |  |  |  |  |  |  |  |  |  |  |  | 4 | 0.499 | 97.77 |
|  |  | x |  |  |  |  |  |  |  |  |  |  |  |  |  |  | 3 | 0.509 | 100.3 |
|  |  |  |  | x |  |  |  |  |  |  |  |  |  |  |  |  | 3 | 0.526 | 105.0 |
|  |  |  |  |  |  |  |  |  |  |  |  |  |  |  |  |  | 2 | 0.530 | 105.6 |
| Wet bout duration in darkness (mins)* | 64 | x |  | x | x |  | x |  |  |  |  |  |  | x |  |  | 8 | 0.729 | 145.6 |
|  |  |  |  | x |  |  | x |  |  |  |  |  |  |  |  |  | 5 | 0.732 | 144.8 |
|  |  |  |  |  |  |  | x |  |  |  |  |  |  |  |  |  | 4 | 0.740 | 146.1 |
|  |  | x |  | x | x |  |  |  | x |  |  |  |  | x |  |  | 7 | 0.744 | 147.2 |

Table S5 continued

|  |  | **Predictor variables** | | | | | | | | | | | | | | |  |  |  |
| --- | --- | --- | --- | --- | --- | --- | --- | --- | --- | --- | --- | --- | --- | --- | --- | --- | --- | --- | --- |
| **Response variable** | **n** | Age | Age^2^ | Sex | Stage | Species | Year | Age:Sex | Age^2^:  Sex | Age:  Stage | Age^2^:  Stage | Age:  Species | Age^2^:  Species | Sex:  Stage | Sex:  Species | Stage:  Species | **df** | **Loocv** | **AICc** |
|  |  | x |  | x | x |  |  |  |  |  |  |  |  | x |  |  | 6 | 0.774 | 146.8 |
| Prop daylight wet (%) | 130 | x | x | x |  | x | x | x | x |  |  |  |  |  |  |  | 13 | 0.514 | 199.5 |
|  |  |  |  |  |  | x | x |  |  |  |  |  |  |  |  |  | 8 | 0.518 | 199.3 |
|  |  | x | x | x |  |  | x | x |  |  |  |  |  |  |  |  | 12 | 0.519 | 203.5 |
|  |  |  |  |  |  |  | x |  |  |  |  |  |  |  |  |  | 7 | 0.524 | 203.6 |
|  |  | x | x | x |  |  |  | x | x |  |  |  |  |  |  |  | 7 | 0.553 | 219.9 |
| Prop darkness wet (%) | 128 |  |  | x | x | x | x |  |  |  |  |  |  |  |  | x | 11 | 0.815 | 315.4 |
|  |  |  |  |  |  | x | x |  |  |  |  |  |  |  |  |  | 8 | 0.815 | 315.4 |
|  |  |  |  |  |  |  | x |  |  |  |  |  |  |  |  |  | 7 | 0.827 | 318.6 |
|  |  |  |  | x | x | x |  |  |  |  |  |  |  |  | x | x | 7 | 0.907 | 341.4 |
|  |  |  |  | x | x | x |  |  |  |  |  |  |  |  |  | x | 6 | 0.909 | 341.7 |
